# Induction of Fibrosis and Autophagy in Kidney Cells by Vinyl Chloride

**DOI:** 10.3390/cells8060601

**Published:** 2019-06-17

**Authors:** Yung-Ho Hsu, Hsiao-Chi Chuang, Yu-Hsuan Lee, Yuh-Feng Lin, Yu-Jhe Chiu, Yung-Li Wang, Mai-Szu Wu, Hui-Wen Chiu

**Affiliations:** 1 Division of Nephrology, Department of Internal Medicine, Shuang Ho Hospital, Taipei Medical University, New Taipei City 23561, Taiwan; yhhsu@s.tmu.edu.tw (Y.-H.H.); linyf@s.tmu.edu.tw (Y.-F.L.); chiuyj2002@gmail.com (Y.-J.C.); 2 Department of Internal Medicine, School of Medicine, College of Medicine, Taipei Medical University, Taipei 11031, Taiwan; 3 School of Respiratory Therapy, College of Medicine, Taipei Medical University, Taipei 11031, Taiwan; chuanghc@tmu.edu.tw; 4 Division of Pulmonary Medicine, Department of Internal Medicine, Shuang Ho Hospital, Taipei Medical University, New Taipei City 23561, Taiwan; 5 School of Public Health, College of Public Health, Taipei Medical University, Taipei 11031, Taiwan; 6 Department of Food Safety/Hygiene &Risk Management, College of Medicine, National Cheng Kung University, Tainan 70430, Taiwan; bmm175@hotmail.com; 7 Graduate Institute of Clinical Medicine, College of Medicine, Taipei Medical University, Taipei 11031, Taiwan; cetuspower@gmail.com

**Keywords:** vinyl chloride, fibrosis, autophagy, kidney

## Abstract

Vinyl chloride (VC) is a noninfective occupational risk factor. It is found in industrial chemicals, volatile organic compounds, cigarette smoke ingredients, etc. It is a kind of toxic gas that causes many diseases. VC exposure causes an increased risk of liver fibrosis and can result in angiosarcoma of the liver. Previous studies have shown that high-doses of VC exposure in mice resulted in acute death with marked tubular necrosis of the renal cortex. In this study, we assessed the nephrotoxicity of VC in vitro and in vivo. As a result, we demonstrated that VC induced fibrosis-associated protein expression, such as connective tissue growth factor (CTGF), plasminogen activator inhibitor-1 (PAI-1) and collagen 1, and autophagy-associated protein expression, such as Beclin 1 and LC3-II, in kidney cells. The beclin1 siRNA experiments found that autophagy inhibited VC-induced fibrosis. Blood urea nitrogen (BUN) and creatinine levels were increased after VC treatment. Furthermore, VC caused glomerulosclerosis and tubular injury in mouse kidney tissues. Kidney tissue sections showed that VC induced fibrosis and autophagy in mouse kidney tissues. In summary, the results of VC-induced fibrosis suggest that autophagy plays an important role in kidney damage. VC may cause nephrotoxicity, and the results illustrate the importance of considering the toxicological hazards of VC in kidney cells.

## 1. Introduction

Vinyl chloride (VC) is an industrial chemical with many applications, but it is mainly used in the manufacturing of polyvinyl chloride [1]. It is a kind of toxic gas that results in systemic toxicity [2,3]. VC is also a decomposition product form volatile organic compounds (VOC) and contaminates groundwater [4]. Additionally, cigarette smoke ingredients (CSI) contain VC [5]. After all, VC is a noninfective occupational risk factor that causes diseases [6]. Most of studies show that VC exposure can increase the risk of developing liver fibrosis [7,8], and periportal fibrosis [9]. Therefore, VC has been suggested to be a carcinogenic factor both in animals and in humans, which mainly results in angiosarcoma of the liver [10,11,12]. Few studies have reported a significant prevalence of lung cancer [13], adrenal gland angiosarcoma [14], and brain injury [15] among exposed workers. In addition, VC results in a 10-fold increase in hepatobiliary cancer mortality and a 13-fold increase in mortality in those exposed to VC for more than 16 years [16]. The relative risk of hepatocellular carcinoma increases in a similar pattern with angiosarcoma of the liver among exposed workers [17]. VC affected hepatic function or fibrosis index in school-aged children living near a petrochemical plant [18]. VC exposure also increases cardiovascular risk [19]. In an animal model, VC extensively caused morphological changes in the respiratory tract, ceruminous glands, brain, kidneys, heart, and spleen in the rat [20]. VC induces several tumor types among several species [21] and has been shown to induce lung cancer in animals [22]. VC also results in pararenal hemangiosarcoma in mice [23] and neural tube defects (NTDs) in embryonic mouse brain tissue [24]. Moreover, all the animals that inhaled VC showed a series of parenchymal lesions and swelling of the kidney parenchyma, assuming a pattern of tubulonephrosis [25].

Chronic kidney disease (CKD) arises from many diverse disease pathways that irreversibly alter the structure and function of the kidneys, causing a chronic reduction in kidney function and chronic kidney damage, such as fibrosis [26]. In an animal model, CKD was shown to follow acute kidney injury (AKI) caused by folic acid and aristolochic acid [27]. Folic acid-induced AKI seem to be a model for early fibrosis and CKD [28]. Survivors of AKI have up to a 28-fold are at higher risk for CKD than healthy individuals [29,30]. Previous studies showed that VC resulted in acute death with marked tubular necrosis of the renal cortex when mice were exposed to high doses of VC [31]. Other studies have shown that autophagy may attenuate fibrosis [32]. Autophagy is a conserved and important ‘‘self-cleansing” pathway [33]. In the kidney, it maintains homeostasis of the glomeruli and tubules [34] and has been implicated in various types of kidney injuries [35,36], aging [37,38] and diseases [39,40,41,42]. Autophagy is absolutely necessary for stress adaption in kidney injuries as it removes protein aggregates and injury organelles and promotes cell survival [43]. However, autophagy can also promote cell death by autophagic cell death or by enhancing apoptosis [44]. Studies have shown that carbon monoxide [45] and cigarette smoke [46] activate autophagy. We found VC had many studies on the liver and fewer studies on the kidney. Theoretically, we suppose VC is systemic toxicity, and does not only result in liver damage. In this study, we tested whether VC induced autophagy in kidney cells. As a result, we found that VC induced autophagy and fibrosis in a human kidney cell (HK-2). Furthermore, VC-induced autophagy inhibited fibrosis. Therefore, autophagy may play a protective role. VC affected kidney function and caused glomerular and tubulointerstitial injury in a mouse model.

## 2. Materials and Methods

### 2.1. Cell Line and Cell Culture Conditions

The human kidney proximal tubular epithelial cell line HK-2 was obtained from the American Type Culture Collection (ATCC, Manassas, VA, USA) and maintained in Keratinocyte-SFM medium (Invitrogen, Carlsbad, CA, USA). Cells were incubated at 37 °C with 5% CO_2_ and cultivated every two or three days.

### 2.2. Cell Viability Assay

Cell viability testing was performed using a sulforhodamine B (SRB, Sigma-Aldrich, St. Louis, MO, USA) assay. SRB dye bonded protein, the amount of dye extracted was standard for the number of cells. Briefly, cells (1 × 10^4^/well) were seeded in a 96-well culture plate and incubated overnight at 37 °C. The cells were fixed with 200 μL of ice-cold 10% trichloroacetic acid (TCA, Sigma) at 4 °C for at least 1 h or overnight. The TCA was removed, and the cells were washed two times with distilled water. After 10 min of air drying, 200 μL of 0.1% sulforhodamine in 1% acetic acid (Sigma) was added, and the cell suspension was incubated at 25 °C for 1 h. Cells were rinsed two times with 1% acetic acid and oven dried at 60 °C for 20 min. Finally, the adhered cells were dissolved in 200 μL 20 mM Tris base (Sigma), and the plate was shaken for 30 min. The absorbance of the cell suspension was measured at a wavelength of 562 nm in an ELISA reader. 

### 2.3. siRNA Knockdown

HK-2 cells were seeded at a density of 3 × 10^5^ cells/well in 6-cm plates overnight without or with beclin 1 siRNA (4392420, Thermo Fisher Scientific, Waltham, MA, USA) according to Mirus transfection protocol (TransIT-X2^®^, Mirus, Madison, WI, USA). Briefly, OptiMEM (Invitrogen) mixed siRNA and TransIT-X2 reagents gently and incubated at room temperatrues for 30 min. A siRNA complex mixture was placed drop-to-drop in each well for 24 h. For VC induction, the cells were induced without VC or with VC at concentrations of 4 μg/mL for 24 h after siRNA incubation.

### 2.4. Western Blot Analysis

Proteins (20–50 µg) were boiled for 5 min in SDS sample buffer (62.5 mM Tris (pH 6.7), 1.25% SDS, 12.5% glycerol, and 2.5% β-mercaptoethanol). Proteins isolated from the cells and HR Pre-Stained Protein Marker 10–170 kDa (BIOTOOLS, New Taipei City, Taiwan) were loaded onto SDS gels and subjected to electrophoresis. After transfer to a PVDF membrane, the proteins on the membrane were incubated with antibodies against Beclin 1 (3738, Cell signaling, Beverly, MA, USA), LC3 (4108, Cell signaling), collagen 1 (14695-1-AP, Proteintech, Rosemont IL, USA), CTGF (23936-1-AP, Proteintech), PAI-1 (11907, Cell signaling) and GAPDH (60004-1-1 g, Proteintech). Immunoreactive bands were visualized using an enhanced chemiluminescence system (Amersham, Little Chalfont, United Kingdom). Protein expression was quantified by ImageQuant version 5.1 (GE Healthcare, Waukesha, WI, USA).

### 2.5. Animal Model

Six-week-old BALB/C male mice were obtained from National Laboratory Animal Center in Taiwan and were maintained in compliance with the institutional policy. All animal procedures were approved by the Institutional Animal Care and Use Committee at Taipei Medical University. VC was diluted in saline and dropped 50 μL into the mouse nose drop by drop with a low dose (1 ng/mL) and a high dose (200 ng/mL) five times per week and samples were harvested in 1, 2 and 3 weeks (5 mice per group). Mice were humanely euthanized at the end of the experiments and kidneys were obtained for histological analysis.

### 2.6. Biochemical Evaluation

Whole blood samples from normal or treated mice were collected by intracardiac puncture. Then, the blood samples were centrifuged at 2000× *g* for 20 min to separate the serum. Biochemical tests included blood urea nitrogen (BUN) levels and creatinine levels. 

### 2.7. Histological Analysis

The kidney tissues were fixed in 10% formalin (Sigma). After 3 days, the tissues were sectioned using a microtome and stained with hematoxylin and eosin (H&E) (Sigma) for histological analyses. The glomerulosclerosis and tubular injury rates were calculated. The glomerulosclerosis score (GS) per kidney (five mice per group) was determined in 50 glomeruli per mouse based on a scale from 0 to 4. The GS was determined as follows: Grade 0, normal glomeruli; Grade 1, presence of mesangial expansion/thickening of the basement membrane; Grade 2, mild/moderate segmental hyalinosis/sclerosis involving less than 50% of the glomerular tuft; Grade 3, diffuse glomerular hyalinosis/sclerosis involving >50% of the tuft; Grade 4, diffuse glomerulosclerosis with total tuft obliteration and collapse. The tubular injury rate of 20 contiguous fields per kidney (five mice per group) was examined. The severity of tubular damage was graded from 0 to 5 according to tubular changes, such as tubular dilatation, loss of brush borders and flattening of the tubular epithelium. The tubular damage index (TDI) was graded as follows: 0, normal; 1, area of tubular dilation and attenuated brush border involving <10%; 2, lesion area between 10 and 20%; 3, lesion area between 20 and 30%; 4, lesion area between 30 and 40%; and 5, lesions involving >40% of the field. The glomerulosclerosis and tubular injury rates were calculated in a blinded manner.

### 2.8. Immunohistochemical (IHC) Staining Analysis

The kidney section was placed in an oven (56 °C) 1 h after the wax was dissolved. The following procedures were used for dewaxing: samples were washed twice with xylene (Sigma) for 5 min, washed twice with 100% alcohol (Sigma) for 5 min, washed twice with 95% ethanol for 5 min, washed twice with 75% alcohol (Sigma) for 5 min, soaked in MQ water for 5 min and other procedures. For immunostaining, samples were boiled with citrate buffer solution (0.01 M, pH 6.0) for 10 min. Samples were then washed twice with PBS for three min, soaked in 3% H_2_O_2_/methanol for 10 min, and washed three times with PBS for 5 min. A background eraser was applied for 15 min, followed by treatment with CTGF (23936-1-AP, Proteintech) or LC3 (PM306, MBL) (O/N). Specimens were washed with PBS twice for three min, covered with Trekkie Universal Link and incubated for 20 min, washed with PBS twice for three min, covered with poly-HRP reagent for 20 min and washed with PBS twice for three min. A DAB coloring agent was then added. After coloring, finished specimens were placed into MQ water to terminate the reaction. Hematoxylin was also used as contrast dye, and specimens were rinsed in running tap water for ten min. Finally cover glue was added to the specimen slide and it was covered with a cover slip. Once the glue solidified, the specimen preparation was completed, and specimens were observed using an optical microscope. For massion staining, trichrome stain kit (ScyTek, Logan, UT, USA) was used and according to protocol.

### 2.9. Statistical Analysis

Data were analyzed by SPSS (SPSS Software, CA, San Diego, USA) and expressed as the mean ± SD. Statistical significance between groups was determined by a two-tailed Student’s t-test. Comparisons within three groups were analyzed by analysis of variance (ANOVA). Significance was determined at *p* < 0.05.

## 3. Results

### 3.1. VC Affected Cell Viability and Induced Fibrosis and Autophagy Reactions in HK-2 Cells

**Figure 1:**
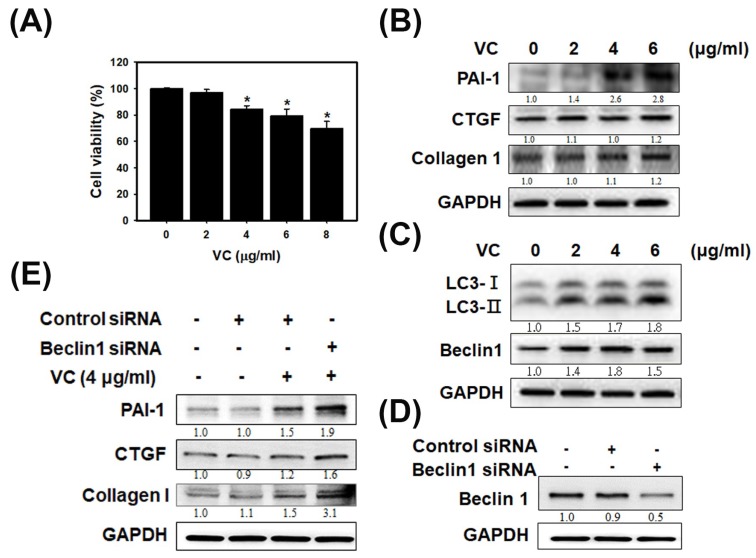
Vinyl chloride (VC) affected cell viability in HK-2 cells and induced fibrosis and autophagy reactions. (**A**) HK-2 cells were seeded at a density of 1 × 10^4^ cells/well in 96-well plates overnight. HK-2 cells decreased cell viability without VC or with VC at concentrations of 2, 4, 6 and 8 μg/mL. * *p* < 0.05 compared with untreated samples. Data are presented as the mean ± SD of three independent experiments. * *p* < 0.05 compared with untreated samples as determined by ANOVA. (**B**) HK-2 cells were seeded at a density of 1 × 10^6^ cells/well in 6-well plates overnight without VC or with VC at concentrations of 2, 4 and 6 μg/mL for 24 h; protein lysates were harvested. Immunoblot of PAI-1, CTGF and Collagen 1 for fibrosis expression in HK-2 cells after cells were treated or not. Glyceraldehyde-3-phosphate dehydrogenase (GAPDH) served as a protein-loading control. (**C**) Immunoblot of LC3 and Beclin 1 in autophagy expression in HK-2 cells after cells were treated or not treated. (**D**) Immunoblot of Beclin1 for siRNA knockdown in HK-2 cells. (**E**) Immunoblot of PAI-1, CTGF and Collagen 1 for fibrosis-related protein expression in HK-2 cells. HK-2 cells were seeded overnight with or without beclin 1 siRNA for 24 h. Then, the cells induced with or without VC at concentrations of 4 μg/mL for 24 h. The quantification of immunoblot intensity is represented by the ratio to untreated samples.

As shown in Figure 1A, VC at 8 μg/mL caused cell death in 30% of HK-2 cells as determined by SRB staining. The results indicated that VC did not induce severe cell death in kidney cells. Next, fibrosis and autophagy were observed in HK-2 cells exposed to VC at 2, 4 and 6 μg/mL (Figure 1B,C). Immunoblotting showed that the expression of plasminogen activator inhibitor type 1 (PAI-1), connective tissue growth factor (CTGF) and collagen 1 increased with fibrosis induction at 6 μg/mL VC (Figure 1B). In particular, PAI-1 expression significantly increased at 4 and 6 μg/mL VC and the PAI-1/GAPDH ratio increased to 2.8. Immunoblotting also showed that the expression of LC3 and Beclin 1 associated with autophagy were clearly increased at 2, 4 and 6 μg/mL VC in HK-2 cells; the LC3II/GAPDH ratio increased to 1.8 and the Beclin 1/GAPDH ratio increased to 1.5 (Figure 1C). After treatment with beclin 1 siRNA, the beclin 1 /GAPDH ratio decreased (Figure 1D), and after being exposed to VC at 4 μg/mL, the PAI-1/GAPDH ratio increased to 1.9, the CTGF/GAPDH ratio increased to 1.6 and the Collagen 1/GAPDH ratio increased to 3.1. Beclin1 siRNA experiments were the link between autophagy and fibrosis (Figure 1E). Figure 1 showed that VC-induced fibrosis and autophagy can inhibit fibrosis in human kidney cells.

### 3.2. VC Increased BUN and Creatinine Levels in An In Vivo Model

**Figure 2:**
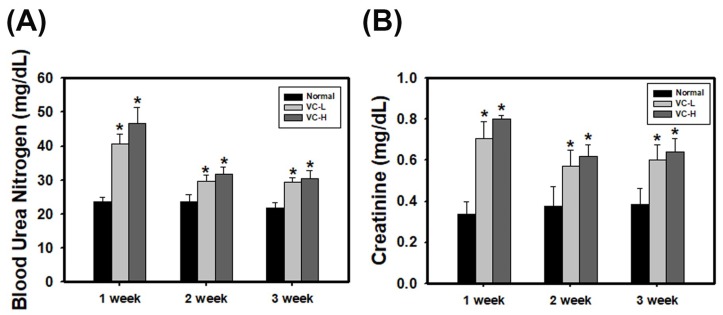
VC increased blood urea nitrogen (BUN) and creatinine levels in mouse serum. VC at a low dose (1 ng/mL) and a high dose (200 ng/mL) was dropped 50 μL onto the noses of six-week-old BALB/C male mice five times per week. Blood was harvested at 1, 2 and 3 weeks after treatment. The blood biochemistry of (**A**) BUN and (**B**) creatinine were tested and analyzed. VC-L indicates a low dose of VC. VC-H indicates a high dose of VC. Data are presented as the mean ± SD. * *p* < 0.05 compared with normal samples as determined by ANOVA.

We further investigated VC exposure in a mouse model. Previously, studies rarely showed that VC affected the kidneys. As a result, a low dose (1 ng/mL) and a high dose (200 ng/mL) of VC were dropped 50 μL onto the noses of six-week-old BALB/C male mice five times per week, and blood was harvested at 1, 2 and 3 weeks. Blood biochemistry showed blood urea nitrogen (BUN) (Figure 2A) and creatinine levels increased after 1 week of VC treatment, regardless of the dose (Figure 2B). In particular, BUN levels at 1 week were significantly increased up to 40.61 and 46.82 mg/dL in a low dose and a high dose mouse, respectively, compared to normal mice (23.69 mg/dL). Creatinine at 1 week was significantly increased up to 0.7071 and 0.8014 mg/dL in a low dose and a high dose mouse, respectively, compared to normal mice (0.3363 mg/dL). The results showed that BUN and creatinine levels were increased after VC treatment regardless of the dose.

### 3.3. VC Increased Glomerulosclerosis and Tubular Injury in Mouse Kidney Tissues

**Figure 3:**
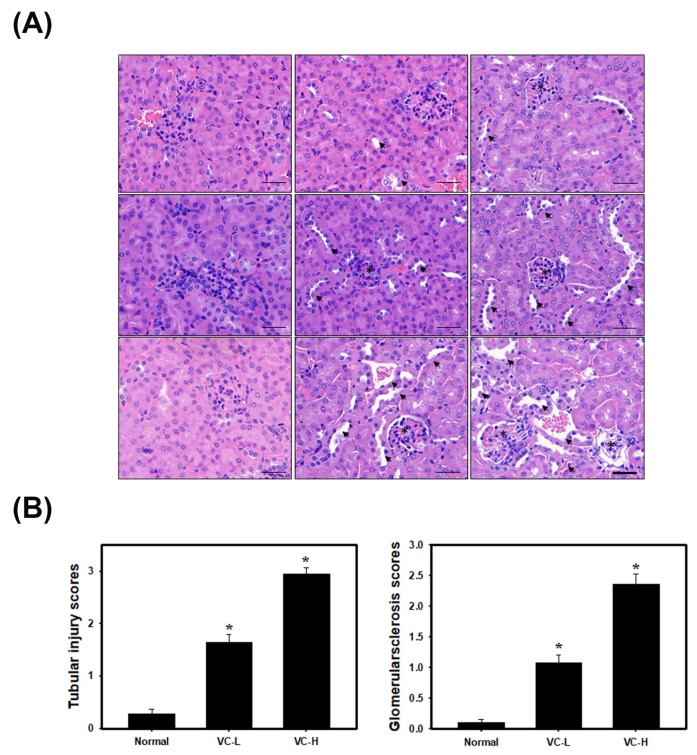
VC increased glomerular and tubulointerstitial injury in mouse kidney tissues. VC at a low dose (1 ng/mL) and a high dose (200 ng/mL) was dropped 50 μL onto the noses of BALB/C male mice 5 times per week for 3 weeks. Kidneys were harvested for paraffin sectioning at 1, 2 and 3 weeks. (**A**) H&E staining. Arrows show tubular injury. Stars show glomerulosclerosis. (**B**) Glomerulosclerosis and tubular injury scores were classed from 1 to 5 and calculated. VC-L indicates a low dose of VC. VC-H indicates a high dose of VC. Data are presented as the mean ± SD. * p < 0.05, compared with normal samples as determined by ANOVA. Bar = 50 μm.

As shown in Figure 3, H&E staining was observed (Figure 3A). The glomerulosclerosis and tubular injury scores were further calculated (Figure 3B) after mice were exposed to VC at a low dose (1 ng/mL) and a high dose (200 ng/mL) 5 times per week for 3 weeks. The glomerulosclerosis injury scores further increased (Figure 3B) up to 1.08 and 2.36 in a low dose and a high dose mouse compared to normal mice (0.108). The tubular injury scores further increased (Figure 3B) up to 1.65 and 2.95 in a low dose and a high dose mouse compared to normal mice (0.28). Figure 3 shows VC-induced glomerulosclerosis and tubular injury in mouse kidney tissue.

### 3.4. VC Increased Fibrosis and Autophagy Markers in Mouse Kidney Tissues

**Figure 4:**
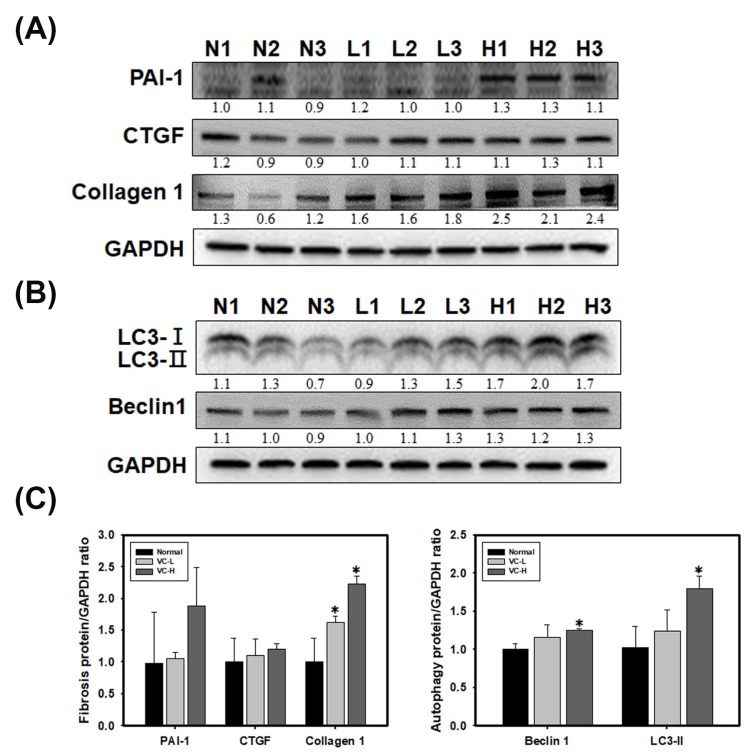
VC increased fibrosis and autophagy markers in mouse kidney tissue. VC at a low dose (1 ng/mL) and a high dose (200 ng/mL) was dropped 50 μL onto the noses of six-week-old BALB/C male mice 5 times per week. The kidneys were harvested after 3 weeks. The kidneys were homogenized and extracted. (**A**) VC-induced fibrosis in the kidneys of six-week-old BALB/C mice. Immunoblots of PAI-1, CTGF and collagen 1 showed fibrosis expression in the kidney after VC treatment or no treatment. (**B**) VC-induced autophagy in the kidneys of six-week-old BALB/C mice. Immunoblotting of LC3 and Beclin 1 revealed autophagy in the kidney after VC treatment or no treatment. (**C**) Protein ratios were calculated and stained after 3 weeks. Data are presented as the mean ± SD. * p < 0.05 compared with normal samples as determined by ANOVA. GAPDH served as a protein-loading control. N indicates normal; L indicates low dose; H indicates high dose; 1, 2 and 3 indicate different mouse kidney tissues.

**Figure 5:**
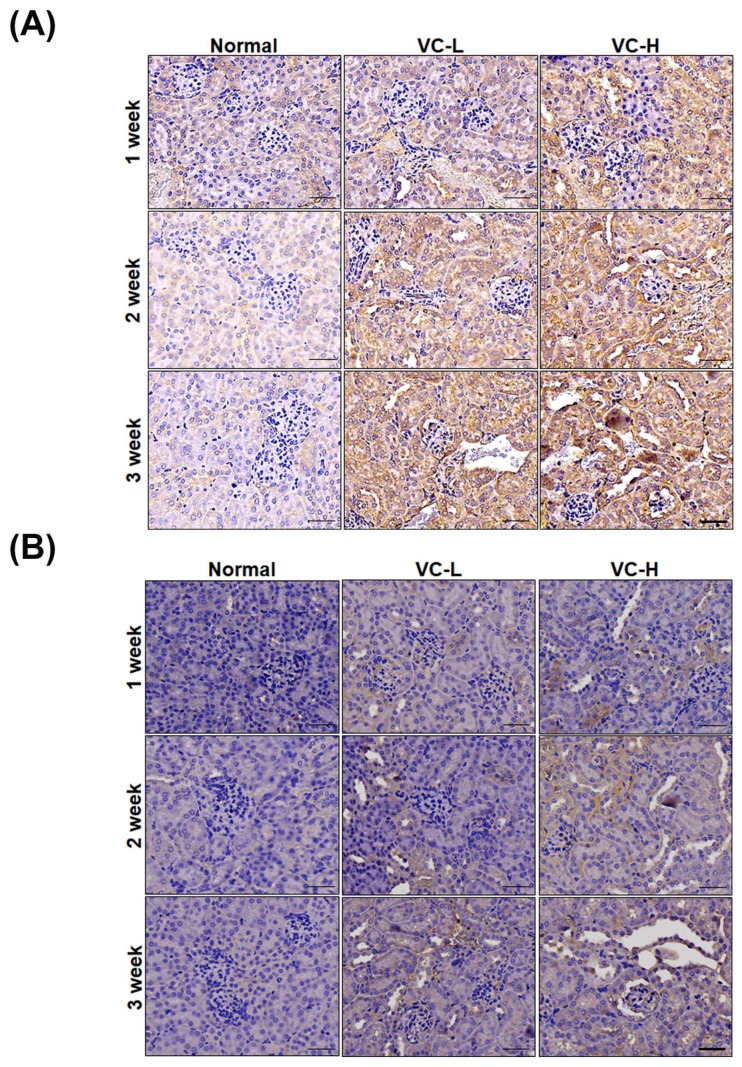

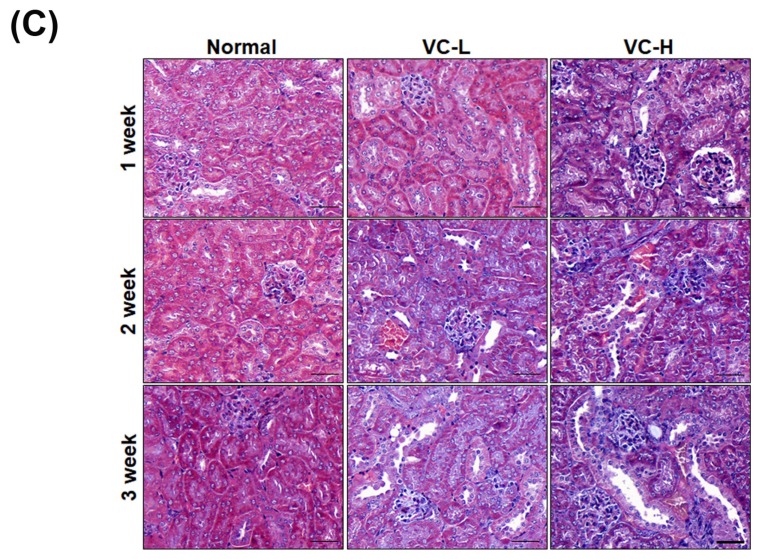
Vinyl chloride increased fibrosis markers and autophagy in mouse kidney tissue. VC at a low dose (1 ng/mL) and a high dose (200 ng/mL) were dropped 50 μL onto the noses of six-week-old BALB/C male mice 5 times per week. Kidneys were harvested for paraffin sectioning after 1, 2 and 3 weeks. Immunohistochemical (IHC) staining for (**A**) CTGF and (**B**) LC3. (**C**) Masson staining. VC-L indicates a low dose of VC. VC-H indicates a high dose of VC. Bar = 50 μm.

As shown in Figure 4, fibrosis and autophagy were found in mouse kidneys after mice were exposed to VC at a low dose and a high dose (Figure 4A,B). Immunoblotting showed that PAI-1, CTGF and collagen 1 increased fibrosis expression (Figure 4A). We found that collagen 1 expression significantly increased up to 2.3 at high doses. Immunoblotting also showed that LC3 and Beclin 1 increased autophagy expression in mouse kidney tissue (Figure 4B). LC3-II expression significantly increased up to 1.79 at high doses. Protein ratios were statistically analyzed and graphed (Figure 4C). As shown in Figure 5, immunohistochemical (IHC) staining showed autophagy and fibrosis in mouse kidneys after mice were exposed to VC at a low dose and a high dose (Figure 5A,B). IHC staining showed that CTGF increased fibrosis expression (Figure 5A). The results indicated that CTGF expression significantly increased at high doses for 3 weeks. In addition, IHC staining showed that LC3 increased autophagy expression (Figure 5B). Masson staining showed that collagen increased fibrosis (Figure 5C). Figure 4 and Figure 5 show VC-induced fibrosis and autophagy in mouse kidney tissues.

## 4. Discussion

The previous study showed an increasing risk of developing liver fibrosis [7,8]. Our results showed that VC also induces kidney fibrosis. However, autophagy inhibited fibrosis through the anti-inflammatory pathway [32,47]. We also found that VC induced autophagy and inhibited fibrosis in kidney cells. Our data confirm that VC promoted kidney injury. Therefore, our results showed that a low dose of VC in a mouse is 1 ng/mL, which equates to 1 ppb, and this caused some damage to the kidney. A recent study showed the annual mean VC level at one campus near a petrochemical plant was 2.19 ppb with a maximum level of 165 ppb [48]. That means that the people who live near the petrochemical plant may be exposed to VC at similar levels to the mice in the experiment. Previous studies had demonstrated that the loss of autophagy is required for the initiation of cancer [49]. Additionally, VC was a carcinogenic factor in both animals and humans and played a major role in angiosarcoma of the liver [10,11,12]. Some studies had indicated that VC caused DNA mutations. VC can cause Ras mutations [50], K-ras-2 mutations [51], p53 mutations [52,53], and p21 mutations [54]. These studies suggest that future experiments should observe DNA mutations after VC exposure in mouse kidneys. Therefore, the protective role of autophagy in the kidney suggests that inducing autophagy may be a promising therapeutic strategy [55,56]. Autophagy plays an important role in kidney injury. The results of mouse experiments show that BUN and creatinine blood levels are lower after 2 and 3-week VC exposure compared to 1-week exposure. This condition seems like an acute response for mouse kidney. The previous studies also show similar results in that the patients of AKI increased in serum creatinine concentration within 2–7 days, and then decreased for a few weeks [26,57]. CKD is a worldwide health concern, and approximately 10% of people have CKD in high-income and middle-income countries. CKD is mainly caused by diabetes, hypertension, or glomerulonephritis [58]. The criteria of CKD include a decreased glomerular filtration rate (GFR) of less than 60 mL/min per 1.73 m^2^, markers of kidney damage (albuminuria (albumin: creatinine ratio [ACR] ≥30 mg/g), urinary sediment abnormalities, electrolyte or other abnormalities due to tubular disorders, abnormalities in histology, structural abnormalities observed by imaging, history of kidney transplantation), or both for at least 3 months in duration [26]. In Asia, India, and sub-Saharan Africa, CKD from glomerulonephritis and unclear causes are more common. Herbal medicines with nephrotoxic effects are sometimes used, causing the digestion of toxic doses of herbs or interactions with western medicines. Water pollution by heavy metals and soil pollution by organic compounds (including pesticides) have also been implicated in geographically localized epidemics of CKD [58]. Notably, VC is a decomposition product of volatile organic compounds (VOCs) and can contaminate groundwater [4]. In an animal model, VC caused extensive morphological changes in many organs in rats [20]. VC results in pararenal hemangiosarcoma in mice [23]. Moreover, all the animals that inhaled VC showed a series of parenchymal lesions and swelling of the kidney parenchyma, assuming the pattern of tubulonephrosis [25]. Therefore, much evidence implies that VC caused kidney injury. Our results show that VC is associated with kidney fibrosis markers. VC induced glomerular and tubulointerstitial injury in mouse kidney tissue. Our results suggest that VC affects kidney function and causes kidney injury.

## 5. Conclusions

**Figure 6:**
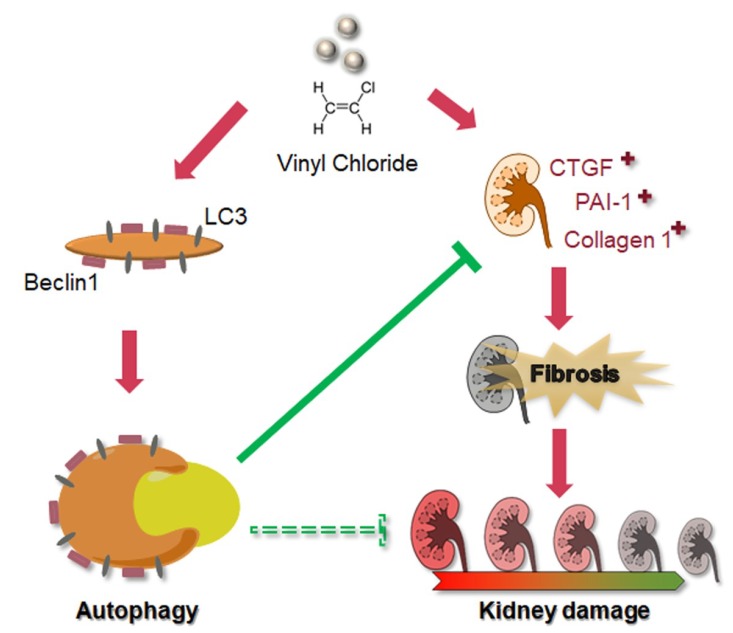
Schematic diagram illustrating the proposed model of VC-induced fibrosis and autophagy. VC induced the fibrosis markers CTGF, PAI-1 and collagen 1. Furthermore, VC increased the autophagy-related protein expression of Beclin 1 and LC3 in kidney cells. However, autophagy may be a stress adaption in VC-induced kidney injury. This is not only stress adaption but VC-induced autophagy inhibited fibrosis. In this study, we found that VC causes kidney damage and induced autophagy to inhibit fibrosis.

As shown in Figure 6, we have demonstrated that VC induced fibrosis markers, including CTGF, PAI-1 and collagen 1, and autophagy-related protein expression, such as Beclin 1 and LC3-II, in kidney cells. VC-induced autophagy inhibited fibrosis. Therefore, autophagy may be a stress adaption in VC-induced kidney injury. BUN and creatinine levels increased after VC treatment. Moreover, VC induced glomerulosclerosis and tubular injury in mouse kidney tissue. Therefore, VC induced renal fibrosis and autophagy that can inhibit fibrosis.
